# Vessel-by-vessel analysis of lower extremity ^18^F-NaF PET/CT imaging quantifies diabetes- and chronic kidney disease-induced active microcalcification in patients with peripheral arterial disease

**DOI:** 10.1186/s13550-023-00951-0

**Published:** 2023-01-17

**Authors:** Ting-Heng Chou, Eleanor T. Rimmerman, Surina Patel, Molly K. Wynveen, Susan N. Eisert, Kumudha Narayana Musini, Sarah A. Janse, Adam J. Bobbey, Timur P. Sarac, Said A. Atway, Michael R. Go, Mitchel R. Stacy

**Affiliations:** 1 Center for Regenerative Medicine, The Research Institute at Nationwide Children’s Hospital, 575 Children’s Crossroad, Columbus, OH WB413343215 USA; 2 Interdisciplinary Biophysics Graduate Program, The Ohio State University, Columbus, OH USA; 3 Center for Biostatistics, The Ohio State University, Columbus, OH USA; 4 Department of Radiology, Nationwide Children’s Hospital, Columbus, OH USA; 5 Division of Vascular Diseases and Surgery, Department of Surgery, The Ohio State University College of Medicine, Columbus, OH USA; 6 Department of Orthopaedics, The Ohio State University College of Medicine, Columbus, OH USA

**Keywords:** Peripheral arterial disease, Diabetes mellitus, Positron emission tomography, Chronic kidney disease, Sodium fluoride

## Abstract

**Background:**

Positron emission tomography (PET)/computed tomography (CT) imaging with fluorine-18 (^18^F)-sodium fluoride (NaF) provides assessment of active vascular microcalcification, but its utility for evaluating diabetes mellitus (DM)- and chronic kidney disease (CKD)-induced atherosclerosis in peripheral arterial disease (PAD) has not been comprehensively evaluated. This study sought to use ^18^F-NaF PET/CT to quantify and compare active microcalcification on an artery-by-artery basis in healthy subjects, PAD patients with or without DM, and PAD patients with or without CKD. Additionally, we evaluated the contributions of DM, CKD, statin use and established CT-detectable calcium to ^18^F-NaF uptake for each lower extremity artery.

**Methods:**

PAD patients (*n* = 48) and healthy controls (*n* = 8) underwent lower extremity ^18^F-NaF PET/CT imaging. Fused PET/CT images guided segmentation of arteries of interest (i.e., femoral-popliteal, anterior tibial, tibioperoneal trunk, posterior tibial, and peroneal) and quantification of ^18^F-NaF uptake. ^18^F-NaF uptake was assessed for each artery and compared between subject groups. Additionally, established calcium burden was quantified for each artery using CT calcium mass score. Univariate and multivariate analyses were performed to evaluate DM, CKD, statin use, and CT calcium mass as predictors of ^18^F-NaF uptake in PAD.

**Results:**

PAD patients with DM or CKD demonstrated significantly higher active microcalcification (i.e., ^18^F-NaF uptake) for all arteries when compared to PAD patients without DM or CKD. Univariate and multivariate analyses revealed that concomitant DM or CKD was associated with increased microcalcification for all arteries of interest and this increased disease risk remained significant after adjusting for patient age, sex, and body mass index. Statin use was only associated with decreased microcalcification for the femoral-popliteal artery in multivariate analyses. Established CT-detectable calcium was not significantly associated with ^18^F-NaF uptake for 4 out of 5 arteries of interest.

**Conclusions:**

^18^F-NaF PET/CT imaging quantifies vessel-specific active microcalcification in PAD that is increased in multiple lower extremity arteries by DM and CKD and decreased in the femoral-popliteal artery by statin use. ^18^F-NaF PET imaging is complementary to and largely independent of established CT-detectable arterial calcification. ^18^F-NaF PET/CT imaging may provide an approach for non-invasively quantifying vessel-specific responses to emerging anti-atherogenic therapies or CKD treatment in patients with PAD.

## Introduction

Lower extremity PAD is a highly prevalent atherosclerotic disease that affects more than 230 million individuals worldwide [1] and is associated with significant morbidity [2], mortality [3], and economic burden [4–6]. Approximately 15–20% of patients with PAD will progress to the severe stage of disease (i.e., chronic limb threatening ischemia, CLTI) [7–9]. Once CLTI is present, 25% of patients will have a major amputation in the first year after diagnosis [10] and approximately 40% of those undergoing major amputation will die within 1 year of surgery [4, 11]. A significant risk factor for developing severe peripheral disease is diabetes mellitus (DM), which increases the incidence of PAD [12] and contributes to a fivefold increased risk of amputation in patients with PAD [13]. Further compounding the risk for many PAD patients is coexisting chronic kidney disease (CKD), which results in worse treatment outcomes in PAD and facilitates vascular calcification through phenotypic switching of vascular smooth muscle cells into osteogenic promoters with calcifying characteristics [14, 15].

Morbidity associated with PAD can be largely attributed to increased calcification of lower extremity arteries [13, 16, 17]. Increasing prevalence of calcification in above- and below-the-knee arteries can impair limb blood flow and muscle perfusion, and the presence of highly calcified lesions below-the-knee can make endovascular access and distal revascularization challenging or impossible, thereby increasing risk of limb loss in PAD patients with severe disease [18–21]. Although studies have shown that calcification of lower extremity arteries contributes to poor outcomes in PAD patients [18, 19, 21, 22], measures of vascular calcification have traditionally been limited to evaluation by CT imaging, which allows for quantification of already established large macroscopic calcium deposits and identifies disease after preventative strategies are not feasible. Therefore, a non-invasive imaging approach that quantifies the early active stages of DM- and CKD-induced vascular microcalcification may allow for improved monitoring and proactive management of PAD patients at increased risk of limb complications and death.

Positron emission tomography (PET) imaging with fluorine-18 (^18^F)-sodium fluoride (NaF) has emerged in recent decades as an approach for identifying the active process of vascular microcalcification in coronary arteries [23–26], and not until recent years have studies begun to translate ^18^F-NaF PET/CT imaging to the lower extremities. These lower extremity studies have shown that femoral artery uptake of ^18^F-NaF is associated with increased levels of plasma total cholesterol and hemoglobin A1c [27] and may predict femoral artery calcium progression [28]. However, clinical investigations have been exclusively limited to assessing ^18^F-NaF uptake in either the femoral or iliac artery and did not specifically focus on evaluating patients with PAD. Further, only in the last year has the potential of ^18^F-NaF PET/CT imaging been highlighted as a possible approach for detecting the active stages of atherosclerosis in below-the-knee arteries [29]. To date, prior imaging studies have not evaluated the contributions of DM and CKD to the active stages of calcific disease progression within individual arteries of PAD patients or performed comprehensive vessel-by-vessel analysis of active microcalcification in the lower extremities of patients with PAD. We hypothesized that ^18^F-NaF PET/CT imaging would allow for non-invasive, comprehensive detection and quantification of DM- and CKD-induced active atherosclerotic disease progression for multiple major arteries of the lower extremities in patients with PAD. Additionally, we hypothesized that PET/CT imaging would elucidate the potential anti-atherogenic actions of statin therapy for patients with PAD. Therefore, in the present study, we performed vessel-by-vessel analysis of ^18^F-NaF PET/CT images acquired in patients with PAD to investigate the contributions of DM and CKD as promoters of arterial microcalcification and the association between statin use and active peripheral microcalcification.

## Methods

### Research subjects

PAD patients (*n* = 48) were recruited from a multidisciplinary vascular surgery/podiatry clinic and prospectively enrolled for static ^18^F-NaF PET/CT imaging of the lower extremities. PAD disease status was determined based on evidence of significant obstructive disease for 1 or multiple lower extremity arteries, as identified by prior abnormal CT angiography, duplex ultrasound, ankle-brachial index (ABI) (< 0.9), and/or toe-brachial index (TBI) (≤ 0.7) [30]. The criteria for DM included fasting plasma glucose measures > 126 mg/dl on two separate occasions, a glycated hemoglobin (HbA1c) ≥ 6.5%, or a 2-h plasma glucose ≥ 200 mg/dl in an oral glucose tolerance test. The presence of CKD and CKD staging for PAD patients was determined through medical chart review and determined by previously published levels of glomerular filtration rate and/or urinary albumin-to-creatine ratio [31].

Healthy control subjects (*n* = 8) were recruited using an Institutional Review Board (IRB)-approved research flier. Healthy subjects were void of PAD, DM, coronary artery disease, cancer, hypertension, and smoking/tobacco use. To ensure that healthy subjects were absent of DM and PAD, all subjects underwent additional screening that included measurement of fasting blood glucose using a finger-prick glucose test, as well as ABI and TBI measures for both lower extremities.

The study protocol was approved by the Nationwide Children’s Hospital IRB, as well as the Radiation Safety Committee, and was in accordance with the guidelines set forth by the Declaration of Helsinki. All individuals provided written informed consent after receiving an explanation of the experimental procedures and potential risks associated with participating in the study.

### PET/CT imaging protocol

All research subjects are reported to the Department of Radiology at Nationwide Children’s Hospital. Subjects were fasted for 4 h, including abstinence from caffeine and alcohol, to standardize patient preparation prior to study involvement. PET/CT imaging of the lower extremities was performed using a hybrid PET/CT system (Discovery 690, GE Healthcare) 75 min after intravenous administration of ^18^F-NaF (dose 342.9 ± 38.3 MBq). The PET imaging field-of-view was set from the upper thigh to the level of the feet. CT images were first acquired with a slice thickness of 3.27 mm, at 120 kV and 85 mA for image registration, attenuation correction, and manual segmentation of arteries of interest. PET imaging was subsequently performed for 7 min per bed position [32]. PET data were reconstructed at 3.27-mm slices with a pixel size of 2.73 mm using the OSEM algorithm with three iterations and 32 subsets, and Gaussian post-reconstruction filter with full width at half maximum of 4 mm.

### PET/CT image analysis

**Fig. 1:**
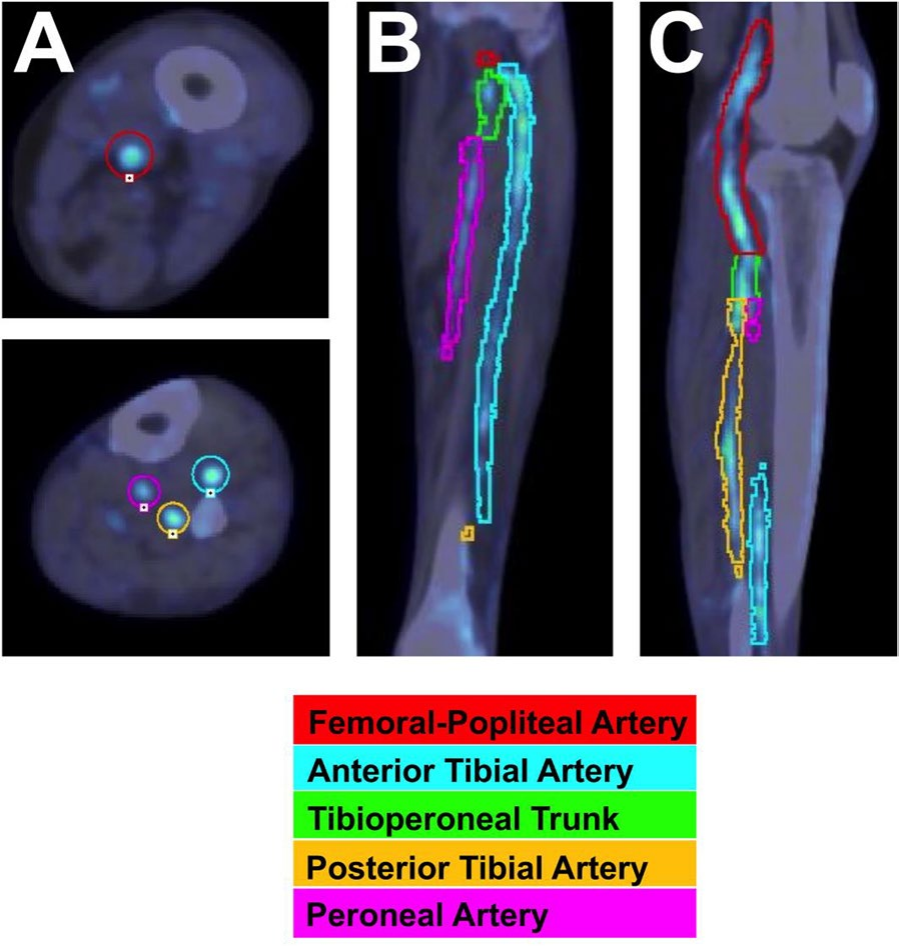
Representative example of lower extremity artery segmentation from CT images acquired in a patient with peripheral arterial disease. ROIs are displayed for arteries of interest in the thigh and calf and shown in **A** axial, **B** coronal, and **C** sagittal planes

PET and CT images were analyzed according to European Association for Nuclear Medicine guidelines for PET imaging of atherosclerosis using commercially available software (PMOD Technologies LLC) [32]. Co-registered PET and CT images were used to define arterial regions of interest (ROIs), which improved identification of peripheral arteries (Fig. 1). Two experienced image analysts manually drew ROIs bilaterally on each axial slice for the femoral-popliteal, anterior tibial, tibioperoneal trunk, posterior tibial, and peroneal arteries to acquire PET-derived measures of ^18^F-NaF uptake for each artery. Arterial ^18^F-NaF uptake values were quantified within each ROI and expressed as standardized uptake values (SUVs). To derive the average maximum SUV (i.e., mean SUV_max_) of each artery, maximum SUVs measured per ROI of each slice were summed and averaged. While SUV_max_ is sensitive to noise, a prior study showed that SUV_max_ provides a good measure of maximum disease burden in a single image slice, and averaging SUV_max_ of all slices (i.e., mean SUV_max_) allowed for less image noise [32]. In addition to calculating mean SUV_max_, the mean SUV_background_ was determined by using the average of five ROIs drawn in the center of the popliteal vein. The average maximum target-to-background ratio (mean TBR_max_) was subsequently calculated by dividing the mean SUV_max_ of each artery by the mean SUV_background_. ^18^F-NaF uptake for each artery of interest was ultimately evaluated and expressed as mean TBR_max_.

To quantify established CT-detectable calcium burden for each artery of interest, each axial CT image was evaluated for calcium burden using the calcium mass score. Specifically, an image intensity threshold of ≥ 130 Hounsfield Units (HUs) was used to detect established arterial calcification for individual axial CT images, and calcium mass score was then quantified for each artery based on the product of the mean density (HU) and volume of calcium per artery [33].

### Ankle- and toe-brachial index

Ankle- and toe-brachial indices (ABI, TBI) were measured using a commercially available portable system (Vicorder, Skidmore Medical Ltd., Bristol, UK). Measurements were acquired for both lower extremities following 20 min of rest in the supine position, as previously described [34].

### Statistical analysis

One-way ANOVA was used to compare ^18^F-NaF uptake values between subject groups for each artery of interest. Unpaired t tests were used to compare demographics between PAD patients and healthy control subjects. Univariate and multivariate linear regression analyses were performed in patients with PAD to identify patient-level factors associated with ^18^F-NaF uptake after adjusting for age, sex, and body mass index. A p value of < 0.05 was considered statistically significant. Estimates are presented with 95% confidence intervals (CI). Statistical analyses were performed using Prism for macOS, version 9.3.0 (GraphPad Software, LLC) or R, version 4.2.0 (R Core Team, 2022).

## Results

### Subject demographics

Standard screening tools that included medical history review and assessment of ABI, TBI, and blood glucose confirmed that all healthy subjects were void of peripheral artery, metabolic, and pulmonary disease and did not have significant risk factors for cardiovascular disease. Conversely, patients with PAD presented with multiple cardiovascular comorbidities and risk factors, which included DM (70.8%), CKD (37.5%), hypertension (83.3%), hyperlipidemia (68.8%), coronary artery disease (29.2%), and current use of tobacco products (31.3%) (Table 1). Of the 18 PAD patients who had CKD, these patients were previously diagnosed with the following stages of CKD: Stage 1 (*n* = 3), stage 2 (*n* = 2), stage 3 (*n* = 6), stage 4 (2), and stage 5 (*n* = 5). Patients with PAD had significantly lower ABIs (*p* = 0.0004) and TBIs (*p* < 0.0001) when compared to healthy control subjects. Additionally, patients with PAD had significantly higher systolic blood pressures (*p* = 0.003) and ages (*p* = 0.01) compared to healthy control subjects. No difference in body mass index existed between subject groups (*p* = 0.13).

**Table 1:** Patient characteristics

	PAD patients, *N* = 48	Healthy controls, *N* = 8
*Demographics*		
Age (years)	60.6 ± 11.8	48.8 ± 11.5
Race		
White	26 (54.2%)	6 (75%)
Black	19 (39.6%)	1 (12.5%)
Middle Eastern	1 (2.1%)	0 (0%)
American Indian/Alaskan	0 (0%)	1 (12.5%)
Refused to answer	1 (2.1%)	0 (0%)
Unknown	1 (2.1%)	0 (0%)
Sex (male)	28 (58.3%)	8 (100%)
Ethnicity		
Hispanic or Latino	1 (2.1%)	1 (12.5%)
Not Hispanic or Latino	47 (97.9%)	7 (87.5%)
Body Mass Index (kg/m^2^)	31.7 ± 8.0	26.6 ± 1.9
Ankle-Brachial Index	0.56 ± 0.38	1.08 ± 0.08
Toe-Brachial Index	0.28 ± 0.24	0.84 ± 0.13
Fasting glucose (mg/dl)	137.2 ± 58.9	95.8 ± 17.0
Systolic blood pressure (mmHg)	137.2 ± 19.8	114.8 ± 14.2
Diastolic blood pressure (mmHg)	74.0 ± 10.8	N/A
Statin use	38 (79%)	1 (12.5%)
*Comorbidities/risk factors*		
Chronic kidney disease	18 (37.5%)	0 (0%)
Coronary artery disease	14 (29.2%)	0 (0%)
Tobacco use	15 (31.3%)	0 (0%)

Values are means ± SD or *n* (%)

N/A = unavailable/not measured

### Group comparisons of vessel-by-vessel active microcalcification

**Fig. 2:**
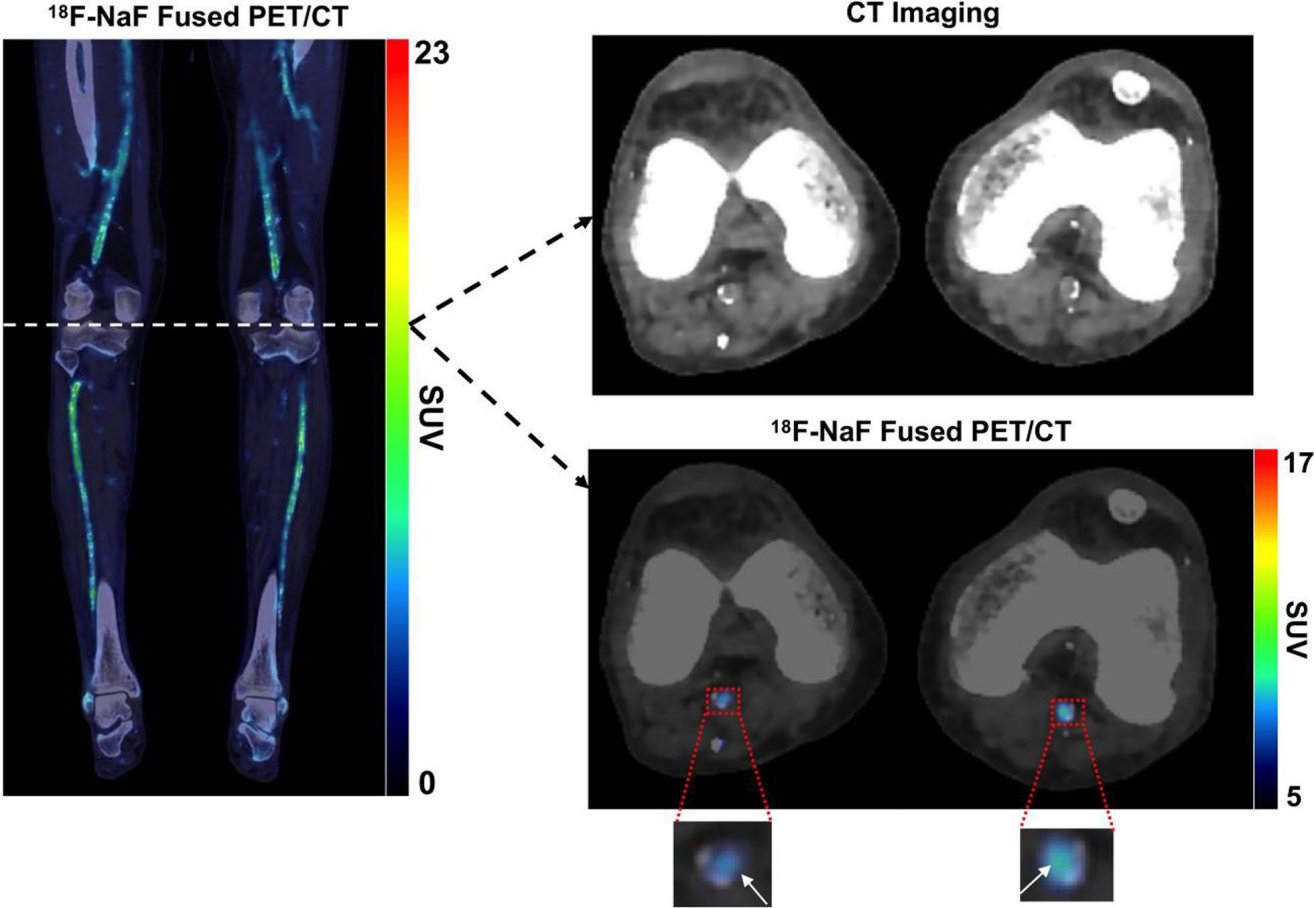
Representative ^18^F-NaF PET/CT imaging of the lower extremities in a patient with PAD and DM. Fused coronal ^18^F-NaF PET/CT imaging of both lower extremities demonstrated heterogenous macrocalcification detected by CT imaging and heterogenous arterial uptake of ^18^F-NaF detected by PET. Focused axial images at the level of the knee further revealed that increased ^18^F-NaF uptake was localized to arterial regions undergoing active microcalcification and were not yet fully detectable by CT imaging (denoted by white arrows)

**Fig. 3:**
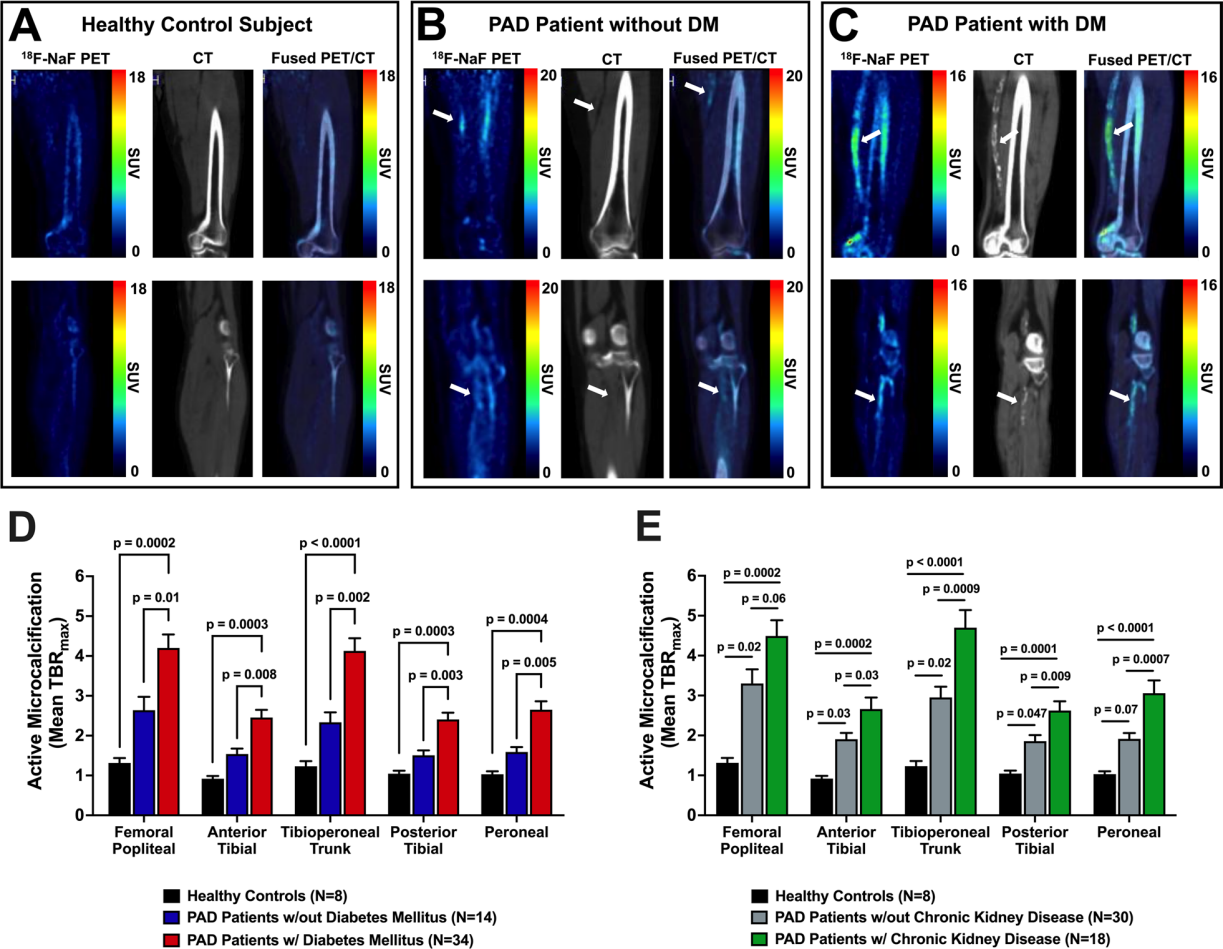
Qualitative and quantitative analyses of lower extremity ^18^F-NaF PET/CT imaging in healthy control subjects and patients with PAD. Representative ^18^F-NaF PET/CT images of a **A** healthy control subject, **B** PAD patient without DM, and **C** PAD patient with DM, which reveal increasing arterial uptake of ^18^F-NaF and established calcium burden with worsening disease status. Quantitative vessel-by-vessel PET/CT image analysis demonstrates **D** DM- and **E** CKD-induced differences in arterial uptake of ^18^F-NaF between patient groups for each lower extremity artery of interest. White arrows denote regions of focal uptake of ^18^F-NaF in patients with PAD. Values represent means ± SEM

Qualitative analysis of ^18^F-NaF PET/CT images non-invasively detected heterogeneous arterial uptake of ^18^F-NaF in both above- and below-the-knee arteries of patients with PAD is shown in Fig. 2. Comparison of ^18^F-NaF uptake between healthy control subjects, PAD patients without DM, and PAD patients with DM demonstrated both qualitative (Fig. 3A–C) and quantitative (Fig. 3D) increases in active arterial microcalcification with increasing disease and the presence of DM. Specifically, ^18^F-NaF uptake (i.e., mean TBR_max_) was significantly higher in PAD patients with DM compared to PAD patients without DM for all five lower extremity arteries of interest (Fig. 3D). Additionally, PAD patients with DM demonstrated significantly higher arterial uptake of ^18^F-NaF for all five arteries of interest when compared to healthy control subjects (Fig. 3D). Similarly, ^18^F-NaF uptake was significantly elevated and different between subject groups in a stepwise manner based on the presence of PAD, as well as the concomitant presence of PAD and CKD (Fig. 3E).

### Univariate and multivariate predictors of active microcalcification in patients with PAD

Univariate linear regression analyses revealed that DM was significantly and positively associated with active arterial microcalcification (i.e., ^18^F-NaF uptake) in patients with PAD for all five lower extremity arteries of interest (Table 2). Specifically, the degree of active microcalcification was increased in PAD patients with DM for the femoral-popliteal (*p* = 0.009), anterior tibial (*p* = 0.005), tibioperoneal trunk (*p* = 0.001), posterior tibial (*p* = 0.002), and peroneal artery (*p* = 0.003). This increased risk for DM-induced active atherosclerotic disease progression remained significant for 4 out of 5 arteries in multivariate analyses after adjusting for age, body mass index, and sex (Table 2).

**Table 2:** Univariate and multivariate analyses of lower extremity microcalcification

	Univariate			Multivariate		
Characteristic	Beta	95% CI	*P* value	Beta	95% CI	*P* value
*Femoral-popliteal artery*						
Age	0.00	− 0.05, 0.05	0.95			
Body mass index	0.05	− 0.01, 0.12	0.12			
Sex (male)	− 0.57	− 1.7, 0.56	0.32			
Diabetes mellitus	1.6	0.42, 2.7	**0.009**	1.5	0.29, 2.8	**0.017**
Chronic kidney disease	1.2	0.08, 2.3	**0.035**	1.1	− 0.01, 2.2	0.053
Statin use	− 1.6	− 2.9, − 0.25	**0.021**	− 1.6	− 2.9, − 0.37	**0.012**
CT calcium mass	0.0004	− 0.00, 0.00	0.064	0.0004	0.00, 0.00	**0.028**
*Anterior tibial artery*						
Age	0.01	− 0.02, 0.03	0.63			
Body mass index	0.05	0.01, 0.09	**0.009**			
Sex (male)	0.35	− 0.28, 1.0	0.27			
Diabetes mellitus	0.92	0.29, 1.5	**0.005**	0.71	0.02, 1.4	**0.044**
Chronic kidney disease	0.76	0.15, 1.4	**0.015**	0.60	0.00, 1.2	0.051
Statin use	− 0.58	− 1.3, 0.18	0.13	− 0.70	− 1.4, 0.01	0.052
CT calcium mass	0.001	− 0.004, 0.002	0.15	0.0009	− 0.00, 0.00	0.18
*Tibioperoneal trunk*						
Age	0.00	− 0.04, 0.05	0.84			
Body mass index	0.09	0.03, 0.15	**0.005**			
Sex (male)	0.53	− 0.54, 1.6	0.32			
Diabetes mellitus	1.8	0.74, 2.8	**0.001**	1.4	0.27, 2.6	**0.017**
Chronic kidney disease	1.8	0.77, 2.7	** < 0.001**	1.5	0.52, 2.5	**0.003**
Statin use	− 1.0	− 2.3, 0.23	0.11	− 1.3	− 2.5, − 0.07	**0.039**
CT calcium mass	0.001	− 0.004, 0.008	0.58	0.0009	− 0.005, 0.007	0.763
*Posterior tibial artery*						
Age	0.00	− 0.02, 0.03	0.78			
Body mass index	0.06	0.03, 0.09	** < 0.0001**			
Sex (male)	0.58	0.03, 1.1	**0.038**			
Diabetes mellitus	0.90	0.34, 1.5	**0.002**	0.60	0.01, 1.2	**0.046**
Chronic kidney disease	0.77	0.24, 1.3	**0.006**	0.57	0.07, 1.1	**0.026**
Statin use	− 0.24	− 0.93, 0.45	0.49	− 0.39	− 1.0, 0.22	0.21
CT calcium mass	0.0009	− 0.001, 0.003	0.434	0.0009	− 0.001, 0.003	0.344
*Peroneal artery*						
Age	0.00	− 0.03, 0.03	0.78			
Body mass index	0.08	0.04, 0.11	** < 0.001**			
Sex (male)	0.40	− 0.29, 1.1	0.25			
Diabetes mellitus	1.1	0.37, 1.8	**0.003**	0.63	− 0.09, 1.3	0.087
Chronic kidney disease	1.1	0.52, 1.8	** < 0.001**	0.91	0.33, 1.5	**0.003**
Statin use	− 0.11	− 1.0, 0.73	0.79	− 0.28	− 1.0, 0.47	0.45
CT calcium mass	0.00003	− 0.003, 0.004	0.987	0.0015	− 0.002, 0.005	0.387

Bold value indicates *P*<0.05

In addition to DM being significantly associated with active arterial microcalcification, CKD was also a significant predictor of active arterial microcalcification in univariate analyses in PAD patients for all five lower extremity arteries of interest (Table 2). In multivariate analyses, CKD remained a determinant of active disease progression for the tibioperoneal trunk (*p* = 0.039), posterior tibial (*p* = 0.026), and peroneal artery (*p* = 0.003) after adjusting for age, body mass index, and sex (Table 2).

Statin use in patients with PAD was found to be significantly and inversely associated with the degree of active arterial microcalcification for 2 out of 5 arteries of interest. Specifically, in univariate analyses, statin use was associated with decreased ^18^F-NaF uptake in the femoral-popliteal artery and tibioperoneal trunk, but was not significantly associated with ^18^F-NaF for the anterior tibial, posterior tibial, or peroneal artery. The association between statin use and active arterial microcalcification remained significant for the femoral-popliteal artery in multivariate analyses after adjusting for age, body mass index, and sex (Table 2).

Established arterial calcification measured by CT imaging (i.e., calcium mass) was significantly associated with active arterial microcalcification (i.e., ^18^F-NaF uptake) in the femoral-popliteal artery in multivariate analyses after adjusting for age, body mass index, and sex (*p* = 0.0276); however, CT-detectable calcium was not significantly associated with the other four arteries of interest below the knee in univariate and multivariate analyses (*p* > 0.05) (Table 2). Further exploratory univariate and multivariate analyses focused on evaluating the association between tobacco use and active microcalcification demonstrated that tobacco use was not significantly associated with ^18^F-NaF uptake for any of the lower extremity arteries of interest (*p* > 0.05 for all arteries, not included in table).

## Discussion

The present study is the first to comprehensively quantify the active stages of atherosclerosis on a vessel-by-vessel basis in patients with PAD, in addition to being the first to non-invasively assess the contributions of DM and CKD to active arterial microcalcification in patients with PAD. Vessel-by-vessel analysis of ^18^F-NaF PET/CT allowed for quantitative evaluation of the active process of atherosclerotic disease and differentiated PAD patients with DM from those without DM and distinguished PAD patients with CKD from those without CKD. Further, univariate and multivariate analyses confirmed that DM and CKD were both significant promoters of active arterial microcalcification for patients with PAD after adjusting for patient age, sex, and body mass index, thus suggesting that ^18^F-NaF PET/CT imaging may provide a sensitive, non-invasive platform for future monitoring of patient-specific vascular responses to DM therapies or CKD treatments.

Previous ^18^F-NaF PET/CT imaging studies have primarily focused on non-invasive evaluation of active microcalcification in the coronary arteries or aorta [35], with limited clinical application of ^18^F-NaF PET/CT to the lower extremities and no prior investigations specifically focusing on patients with PAD. A prior study by Takx et al. [27] evaluated determinants of ^18^F-NaF uptake in the femoral arteries of 68 patients with DM; however, only 24% of participants in this cohort were previously diagnosed with PAD. Takx et al. [27] found that hemoglobin A1c levels were positively associated with ^18^F-NaF uptake in the femoral arteries, thus suggesting a potential role of glucose management in mediating active microcalcification of lower extremity arteries. The present study clarifies and builds on this prior work by demonstrating that DM is significantly associated with ^18^F-NaF uptake in not only the femoral artery, but also in multiple above- and below-the-knee lower extremity arteries in the setting of PAD. This finding related to DM-induced active microcalcification in PAD also builds on prior clinical studies that have revealed through angiographic and hemodynamic assessment that patients with DM have a higher propensity than non-DM patients for femoral-popliteal and below-the-knee vascular disease [13, 17]. In addition to demonstrating a significant positive association between DM and active microcalcification, we found that a clinical diagnosis of CKD, regardless of CKD stage, was significantly associated with an increase in active microcalcification of multiple lower extremity arteries in patients with PAD. This is in agreement with a large body of existing literature that has demonstrated CKD increases prevalence of PAD [36]; however, the present study provides novel insight into CKD-driven pathophysiology in patients with PAD, while prior work by Takx et al. [27] did not find a significant association between statin use and ^18^F-NaF uptake in the femoral artery, the present study revealed that statin use was significantly associated with a reduction in ^18^F-NaF uptake in the femoral-popliteal artery, as well as the tibioperoneal trunk, thus indicating that PET/CT imaging may quantify the protective actions of statin therapy in patients with PAD on a vessel-specific basis. Thus, the present study provides novel insight into PAD pathophysiology and sheds light on the specific contributions of DM, CKD, and statin use to active calcific disease in patients with PAD.

In addition to evaluating the contributions of DM, CKD, and statin use to active microcalcification in lower extremity PAD, univariate and multivariate analyses also revealed that established CT-detectable arterial calcium (i.e., calcium mass) was not significantly associated with active microcalcification (i.e., ^18^F-NaF uptake) for 4 out of the 5 arteries assessed in the present study. This finding supports the central hypothesis that ^18^F-NaF PET/CT imaging detects and quantifies the process of active arterial microcalcification in PAD and is complementary to and largely independent of established arterial calcification detected by CT imaging. Future use of vessel-specific analysis of ^18^F-NaF PET/CT imaging in patients with PAD could assist in non-invasively identifying sites of subclinical active disease that may guide personalized treatment or targeted interventions on a vessel- or lesion-specific basis.

Although ^18^F-NaF PET/CT imaging provided comprehensive vascular assessment of patients with PAD, it should be noted that the total number of patients enrolled in the present study was relatively small. Therefore, expanded application of vascular PET/CT imaging in patients with PAD is warranted to validate the findings of this study and fully elucidate the contributions of DM and CKD as promoters of active microcalcification on a broader scale in the setting of PAD. Evaluation of a larger sample size may also allow for more detailed analysis of how DM and CKD disease severity determine the degree of active arterial microcalcification. In addition to the relatively small cohort of PAD patients, the vessel-by-vessel analysis performed in the present study required time-consuming manual segmentation of the lower extremity arterial network. Future implementation of machine learning methods may assist in streamlining this arterial image analysis approach for broader use within the vascular medicine community to better characterize and non-invasively monitor the pathophysiology of lower extremity PAD.

## Data Availability

The datasets used and/or analyzed during the current study are available from the corresponding author on reasonable request.

## References

[1] Criqui MH, Matsushita K, Aboyans V, Hess CN, Hicks CW, Kwan TW (2021). Lower extremity peripheral artery disease: contemporary epidemiology, management gaps, and future directions: a scientific statement from the American Heart Association. Circulation.

[2] Hirsch AT, Criqui MH, Treat-Jacobson D, Regensteiner JG, Creager MA, Olin JW (2001). Peripheral arterial disease detection, awareness, and treatment in primary care. JAMA.

[3] Barnes JA, Eid MA, Creager MA, Goodney PP (2020). Epidemiology and risk of amputation in patients with diabetes mellitus and peripheral artery disease. Arter Thromb Vasc Biol..

[4] Hirsch AT, Hartman L, Town RJ, Virnig BA (2008). National health care costs of peripheral arterial disease in the Medicare population. Vasc Med.

[5] Jaff MR, Cahill KE, Yu AP, Birnbaum HG, Engelhart LM (2010). Clinical outcomes and medical care costs among medicare beneficiaries receiving therapy for peripheral arterial disease. Ann Vasc Surg.

[6] Huang ES, Basu A, O’Grady M, Capretta JC (2009). Projecting the future diabetes popluation size and related costs for the US. Diabetes Care..

[7] Jelnes R, Gaardsting O, Jensen KH, Baekgaard N, Tonnesen KH, Schroeder T (1986). Fate in intermittent claudication: outcome and risk factors. Br Med J (Clin Res Ed).

[8] Weitz JI, Byrne J, Clagett GP, Farkouh ME, Porter JM, Sackett DL (1996). Diagnosis and treatment of chronic arterial insufficiency of the lower extremities: a critical review. Circulation.

[9] Dormandy J, Mahir M, Ascady G, Balsano F, De Leeuw P, Blombery P (1989). Fate of the patient with chronic leg ischaemia. A review article. J Cardiovasc Surg.

[10] Norgren L, Hiatt WR, Dormandy JA, Nehler MR, Harris KA, Fowkes FGR (2007). Inter-society consensus for the management of peripheral arterial disease. J Vasc Surg.

[11] Duff S, Mafilios MS, Bhounsule P, Hasegawa JT (2019). The burden of critical limb ischemia: a review of recent literature. Vasc Heal Risk Manag.

[12] Beckman JA, Creager MA, Libby P (2002). Diabetes and atherosclerosis: epidemiology, pathophysiology, and management. JAMA.

[13] Jude EB, Oyibo SO, Chalmers N, Boulton AJ (2001). Peripheral arterial disease in diabetic and nondiabetic patients: a comparison of severity and outcome. Diabetes Care.

[14] Anantha-Narayanan M, Sheikh AB, Nagpal S, Jelani Q-A, Smolderen KG, Regan C (2021). Systematic review and meta-analysis of outcomes of lower extremity peripheral arterial interventions in patients with and without chronic kidney disease or end-stage renal disease. J Vasc Surg.

[15] Li Z, Wu J, Zhang X, Ou C, Zhong Z, Chen Y (2019). CDC42 promotes vascular calcification in chronic kidney disease. J Pathol.

[16] Rocha-Singh KJ, Zeller T, Jaff MR (2014). Peripheral arterial calcification: prevalence, mechanism, detection, and clinical implications. Catheter Cardiovasc Interv.

[17] Haltmayer M, Mueller T, Horvath W, Luft C, Poelz W, Haidinger D (2001). Impact of atherosclerotic risk factors on the anatomical distribution of peripheral arterial disease. Int Angiol.

[18] Huang C-L, Wu I-H, Wu Y-W, Hwang J-J, Wang S-S, Chen W-J (2014). Association of lower extremity arterial calcification with amputation and mortality in patients with symptomatic peripheral artery disease. PLoS ONE.

[19] Chowdhury MM, Makris GC, Tarkin JM, Joshi FR, Hayes PD, Rudd JHF (2017). Lower limb arterial calcification (LLAC) scores in patients with symptomatic peripheral arterial disease are associated with increased cardiac mortality and morbidity. PLoS ONE.

[20] Ohtake T, Oka M, Ikee R, Mochida Y, Ishioka K, Moriya H (2011). Impact of lower limbs’ arterial calcification on the prevalence and severity of PAD in patients on hemodialysis. J Vasc Surg.

[21] Guzman RJ, Brinkley DM, Schumacher PM, Donahue RMJ, Beavers H, Qin X (2008). Tibial artery calcification as a marker of amputation risk in patients with peripheral arterial disease. J Am Coll Cardiol.

[22] Smolderen KG, van Zitteren M, Jones PG, Spertus JA, Heyligers JM, Nooren MJ (2015). Long-term prognostic risk in lower extremity peripheral arterial disease as a function of the number of peripheral arterial lesions. J Am Hear Assoc.

[23] Dweck MR, Chow MWL, Joshi NV, Williams MC, Jones C, Fletcher AM (2012). Coronary arterial 18F-sodium fluorid uptake: a novel marker of plaque biology. J Am Coll Cardiol..

[24] Irkle A, Vesey AT, Lewis DY, Skepper JN, Bird JLE, Dweck MR (2015). Identifying active vascular microcalcification by 18F-sodium fluoride positron emission tomography. Nat Commun.

[25] Li Y, Berenji GR, Shaba WF, Tafti B, Yevdayev E, Dadparvar S (2012). Association of vascular fluoride uptake with vascular calcification and coronary artery disease. Nucl Med Commun.

[26] Stacy MR (2019). Radionuclide imaging of atherothrombotic diseases. Curr Cardiovasc Imaging Rep.

[27] Takx RAP, van Asperen R, Bartstra JW, Zwakenberg SR, Wolterink JM, Celeng C (2021). Determinants of 18F-NaF uptake in femoral arteries in patients with type 2 diabetes mellitus. J Nucl Cardiol.

[28] den Harder AM, Wolterink JM, Bartstra JW, Spiering W, Zwakenberg SR, Beulens JW (2021). Vascular uptake on 18F-sodium fluoride positron emission tomography: precursor of vascular calcification?. J Nucl Cardiol.

[29] Eisert SN, Chou TH, Bobbey AJ, Go MR, Stacy MR (2020). Noninvasive detection of active microcalcification in an occlusive peripheral vascular aneurysm using 18F-NaF PET/CT imaging. Clin Nucl Med.

[30] Gerhard-Herman MD, Gornik HL, Barrett C, Barshes NR, Corriere MA, Drachman DE (2017). 2016 AHA/ACC guideline on the management of patients with lower extremity peripheral artery disease: A report of the American College of Cardiology/American Heart Association task force on clinical practice guidelines. J Am Coll Cardiol.

[31] Lamprea-Montealegre JA, Shlipak MG, Estrella MM (2021). Chronic kidney disease detection, staging, and treatment in cardiovascular disease prevention. Heart.

[32] Bucerius J, Hyafil F, Verberne HJ, Slart RHJA, Lindner O, Sciagra R (2016). Position paper of the Cardiovascular Committee of the European Association of Nuclear Medicine (EANM) on PET imaging of atherosclerosis. Eur J Nucl Med Mol Imaging.

[33] Agatston AS, Janowitz WR, Hildner FJ, Zusmer NR, Viamonte M, Detrano R (1990). Quantification of coronary calcium using ultrafast computed tomography. J Am Coll Cardiol.

[34] Teren A, Beutner F, Wirkner K, Loeffler M, Scholz M (2013). Validity, intra- and inter-observer reliability of automated devices for the assessment of ankle brachial index using photo-plethysmography. BMC Cardiovasc Disord.

[35] Tzolos E, Dweck MR (2020). 18F-sodium fluoride (18F-NaF) for imaging microcalcification activity in the cardiovascular system. Arter Thromb Vasc Biol.

[36] Serra R, Bracale UM, Ielapi N, Del Guercio L, Di Taranto MD, Sodo M (2021). The impact of chronic kidney disease on peripheral artery disease and peripheral revascularization. Int J Gen Med.

